# Protocol for systematic reviews of school-based food and nutrition education intervention for adolescent health promotion: Evidence mapping and syntheses

**DOI:** 10.1097/MD.0000000000016977

**Published:** 2019-08-30

**Authors:** Gidyenne Christine Bandeira Silva de Medeiros, Kesley Pablo Morais de Azevedo, Daniel Ángel Garcia, Victor Hugo de Oliveira Segundo, Ádala Nayana de Sousa Mata, Karla Silveria Dias Pinheiro de Siqueira, Anny Karoliny Pinheiro Fernandes, Raquel Praxedes dos Santos, Débora Danielly Barros de Brito Trindade, Clélia de Oliveira Lyra, Grasiela Piuvezam

**Affiliations:** a Department of Nutrition, Federal University of Rio Grande do Norte; b Graduate Program in Public Health, UFRN, Natal, Brazil; c Department of Physiotherapy, San Antonio de Murcia Catholic University, Spain; d Multicampi School of Medical Sciences of RN; e Monte das Gameleiras Municipal Government; f Department of Public Health, UFRN, Natal, Brazil.

**Keywords:** adolescent, education, intervention, nutrition, protocol, school

## Abstract

**Background:**

Nutrition is an important modifiable factor in combating overweight and obesity among adolescents. School has been indicated as an effective environment for influencing eating behavior; however, recent reviews assessing school-based interventions specifically for adolescents are scarce. Therefore, we propose the present systematic review with the aim to comprehensively review the quantitative and qualitative literature on the effects of school-based food and nutrition education interventions on adolescent health promotion through healthy eating habits.

**Methods:**

We will search MEDLINE/PubMed, Embase, Scopus, ERIC, ScienceDirect, Web of Science, Cochrane, LILACS, and ADOLEC. We will include randomized controlled trials (RCT), non-RCT, and controlled before-after studies. Risk of bias will be assessed using the EPOC Risk of Bias Tool for RCT, Risk of Bias in Non-randomized Studies of Interventions (ROBINS-I) tool for non-RCT or controlled before-after, as well as the Critical Appraisal Skills Program (CASP) checklist for qualitative studies. We will analyze the overall strength of the evidence for each outcome using the Grading of Recommendations Assessment, Development and Evaluation (GRADE) tool. Two independent researchers will conduct all evaluations and any disagreements will be consulted with a third reviewer. Data analysis and synthesis will be analyzed by the RevMan 5.3 software. We will conduct the study in accordance with the guideline of the Preferred Reporting Items for Systematic Review and Meta-analyses Protocols.

**Result:**

This review will evaluate the effects of school-based food and nutrition education interventions on adolescent health promotion through healthy eating habits. The primary outcome will be changes in adolescent food consumption. Secondary outcomes will be biological parameters (e.g., body mass index (BMI), waist circumference (WC), body composition, etc); biochemical parameters (e.g., glycemia, triglycerides, total cholesterol, etc); qualitative evidences that support or explain the effect of school-based food and nutrition education interventions on adolescent food consumption.

**Conclusion:**

The findings of this systematic review will summarize the latest evidence of the effects of school-based food and nutrition education interventions on adolescent health promotion. The findings will be an available reference for school-based interventions and other further research.

**Registration:**

PROSPERO CRD42019116520.

## Abstract 2

Supplemental Digital Content is available in the text

## 1 Introduction

The first decades of the 21st century were marked by an increase in the prevalence of overweight and obesity in adolescents. This context becomes worrying from a public health point of view since obesity in the adolescent population can cause breathing difficulties, increased risk of fractures, hypertension, early markers of cardiovascular disease, insulin resistance, and psychological effects. In addition, at this stage of life, obesity is associated with a greater chance of obesity, premature death, and disability in adult life.^[1]^

In this perspective, an important modifiable factor able to combat the rise in prevalence of overweight and obesity and their consequences in this population is nutrition. Corroborating this, scientific evidence shows that changes in diet have major effects on the individual's current and future health.^[2]^

It is appropriate to emphasize that this phase of life is an important time to lay the foundations for health in adult life. It is a time of biological and social change^[3]^ in which, often, the food behavior becomes unhealthy.^[4]^ Adolescents now have more autonomy in their food choices, and therefore intervention strategies must be differentiated and targeted to this audience.

Due to the reciprocal relationship between health and education, the school is an effective environment for health promotion, including to influence the eating behavior of adolescents.^[5,6]^ In this school context, a holistic approach to health promotion can be established, involving families and communities to reinforce health messages outside the school environment.^[7]^

The literature presents systematic reviews and meta-analyses that address school-based food and nutrition education interventions geared mainly for children or for the child-adolescent binomial.^[8–11]^

However, current systematic reviews that evaluate school-based interventions specifically for adolescents, besides being scarce,^[12–15]^ present specific approaches geared towards technology-based methodologies,^[12,13]^ consider only 1 outcome (fruit and vegetable consumption),^[14]^ or they restrict the scope of the research to developed countries and the time of publication.^[15]^

Hence, this work aims to comprehensively review the quantitative and qualitative literature on the effects of school-based food and nutrition education interventions on adolescent health promotion. The following review questions will be considered:

1) What are the effects of school-based food and nutrition education interventions on adolescent food consumption?;
2) What are the effects of school-based food and nutrition education interventions on adolescent biochemical parameters?;
3) What are the effects of school-based food and nutrition education interventions on adolescent biological parameters?;
4) What qualitative evidence explains the success of school-based food and nutrition education interventions on adolescent food consumption?

## 2 Methods

### 2.1 Study registration

This systematic review protocol has been registered on the PROSPERO database (CRD42019116520), based on the Preferred Reporting Items for Systematic Reviews and Meta-Analyses Protocols (PRISMA-P) statement guidelines.^[16]^ This is a literature-based study. Ethical approval is unnecessary.

### 2.2 Eligibility criteria

#### 2.2.1 Types of studies

We will include randomized controlled trials (RCT) (including clustered), non-RCT, or controlled before-after studies that have reported interventions to promote adolescent health through changes in food consumption, biological or biochemical parameters in an intervention group when compared to a control group.

#### 2.2.2 Types of participants

We will include studies that recruited adolescents only. For the purposes of the review, adolescents were defined according to the World Health Organization definition of people aged 10 to 19 years.^[17]^

#### 2.2.3 Types of interventions

We will include studies that have implemented school-based food and nutrition education interventions. Non-school-based comparators—standard, no intervention or other intervention—will be accepted. Studies without a control group but subjectively measured outcomes (self-report, interviews, questionnaires) will be included.

#### 2.2.4 Outcome measures

The primary outcome measures will be changes in adolescent food consumption. The secondary outcome measures will include the changes of biological parameters (e.g., body mass index (BMI), waist circumference (WC), waist-to-height ratio (WHR), total body fat, etc); biochemical parameters (e.g., glycemia, triglycerides, total cholesterol, HDL cholesterol, LDL cholesterol); qualitative evidences that support or explain the effect of school-based food and nutrition education interventions on adolescent food consumption. Studies will be included if they report at least one of the following outcome measures.

#### 2.2.5 Exclusion criteria

We will not include studies that:

1) the participants were adolescents with physical disabilities, intellectual disabilities, endocrine disorders, chronic diseases (cardiovascular diseases, diabetes), and pregnant;
2) the participants consist of children and adolescents, without analysis of the adolescent subgroup;
3) studies that did not describe the methodology of the food and nutrition education intervention;
4) studies that only evaluated nutrients and not food.

### 2.3 Search methods for study identification

The review will be divided into 3 thematic areas: food consumption; biological and biochemical parameters associated with food consumption; qualitative evidences. The evidence from the qualitative research will be used to explain the quantitative findings and will provide a deeper understanding of effective school-based strategies to influence adolescent food consumption.

For each thematic area, the revisions will be carried out in the following stages:

1) apply the broad inclusion and exclusion criteria to the searches in the databases by reading the titles and abstracts;
2) apply the eligibility criteria after reading the full texts of the articles selected in the first stage;
3) evaluate the methodological quality and risk of bias of the articles included in the second stage;
4) qualitative synthesis of data from included studies (narrative synthesis or meta-synthesis);
5) quantitative synthesis (meta-analysis).

#### 2.3.1 Electronic search

A comprehensive search will be performed for relevant studies in the following databases, using the search terms detailed in Appendix 1: MEDLINE (via PubMed), Embase (via OVID), Scopus (via Elsevier), Education Resources Information Center (ERIC), Science Direct (via Elsevier), Web of Science-Main Collection (Clarivate Analytics), Cochrane Central Register of Controlled Studies (CENTRAL), LILACS (via Virtual Health Library), and ADOLEC (via Virtual Health Library). There will be no limitation of time and languages.

#### 2.3.2 Additional search

To ensure comprehensiveness of this research, we will supplement searches by hand-searching in the reference lists of retrieved studies or the relevant reviews.

### 2.4 Study selection and data extraction.

Considering that the studies may be common to the 3 thematic areas of revision, the searches in the databases will be performed together. For all identified studies, at least 2 authors will independently select and review titles and abstracts using the Rayyan web application.^[18]^ Papers which meet the inclusion criteria will be ordered for full review. Any disagreement will be resolved by discussion with a third reviewer.

**Figure 1:**
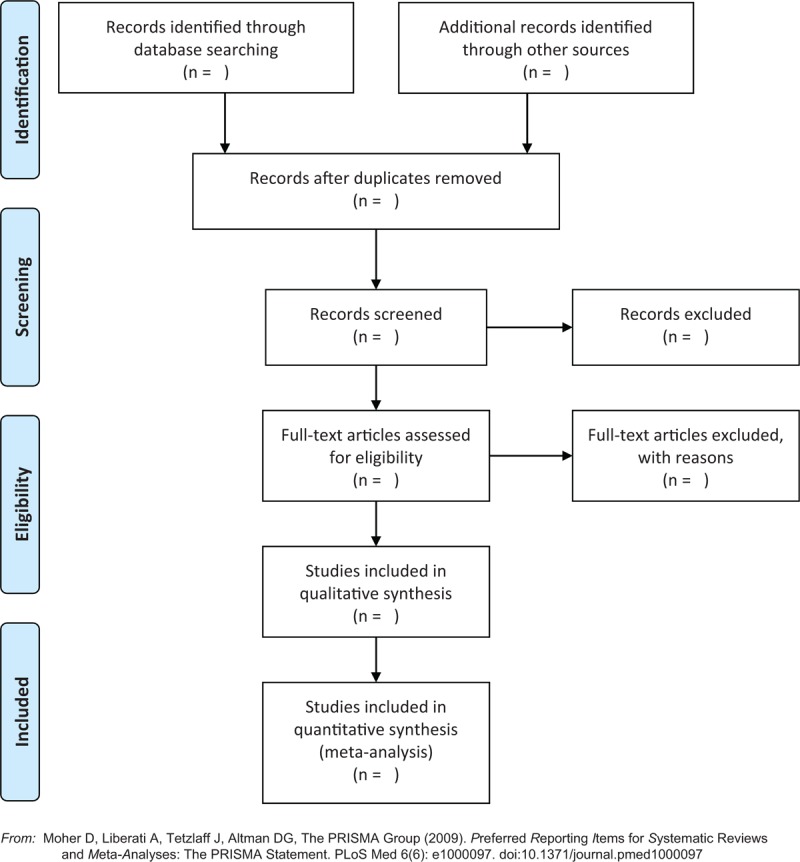
PRISMA flowchart of study selection.

All information on the phases of the selection process will be identified in Figure 1, based on the Preferred Reporting Items for Systematic Reviews and Meta-Analyses (PRISMA).^[19]^

Two reviewers will extract the following information from the selected relevant studies: publication identity (ID), participants’ characteristics, control group, intervention characteristics, dietary assessment, outcome measurements, and analysis methods. The data to be extracted is available in Table 1. Any disagreement will be resolved by discussion and re-examination of the article. A third researcher will be consulted.

**Table 1:**
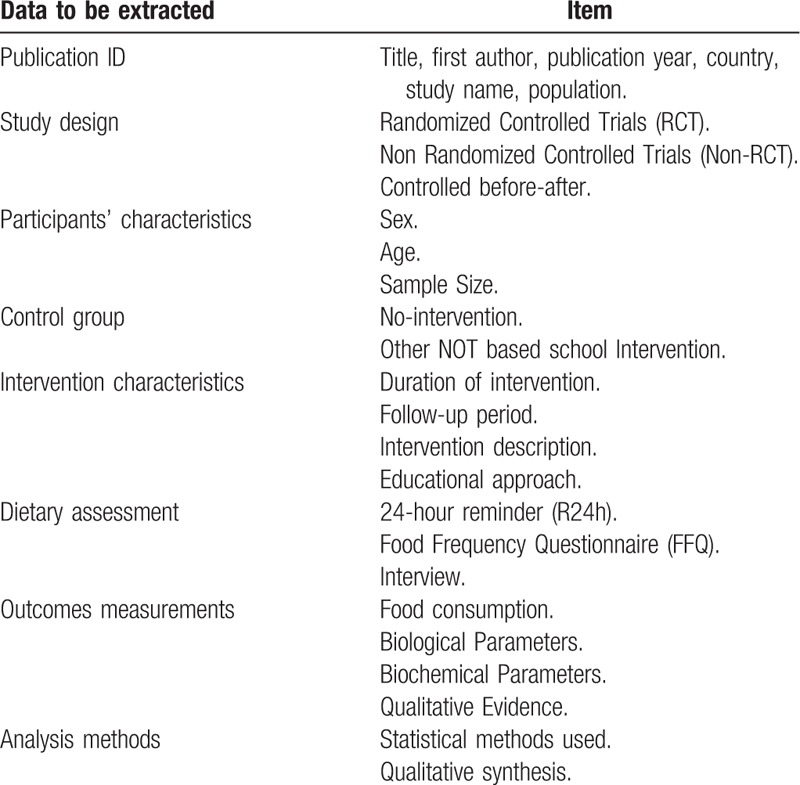
Data extraction table.

### 2.5 Data analysis

#### 2.5.1 Risk of bias in the included studies

Two independent researchers will carry out the evaluation, and when there are doubts or discrepancies, a third researcher will be consulted. The methodological quality of the studies will be assessed using the Revised Cochrane risk-of-bias tool for randomized trials (RoB 2).^[20]^ The following criteria will be assessed in intervention studies: random sequence generation, allocation concealment, blinding of participants, and clinicians and outcome assessment. In addition, incomplete outcome data, selective reporting, funding, and potential for conflicts of interest associated with the individual trials will also be considered. The risk of bias will be rated using predetermined criteria as follows: low, high, or unclear.

For non-RCT and controlled before-after studies, risk of bias will be assessed using the Risk of Bias in Non-randomized Studies of Interventions (ROBINS-I) tool. The ROBINS-I was developed to assess risk of bias in the results of non-randomized studies that compare health effects of 2 or more interventions.^[21]^

For qualitative studies, risk of bias will be assessed using the Critical Appraisal Skills Program (CASP) checklist with 10 questions, 9 addressing quality, and 1 addressing “value” (contribution to existing literature).^[22]^ This checklist is recommended by the Cochrane Collaboration for qualitative literature.^[23]^

We will analyze the overall strength of the evidence for each outcome using the Grading of Recommendations Assessment, Development and Evaluation (GRADE) tool.^[24]^

#### 2.5.2 Statistical analysis

A narrative approach will be used to summarize the effectiveness of the interventions. Food consumption and educational approach of intervention studies will be looked at separately, and if the studies are sufficiently homogeneous, a quantitative synthesis will be undertaken.

Meta-analysis of the included studies will be handled using statistical software (RevMan 5.3). The heterogeneity between trial results will be evaluated using a standard X^2^ test with a significance level 0.05. To assess heterogeneity, we plan to compute the I^2^ statistic, which is a quantitative measure of inconsistency across studies. A value of 0% indicates no observed heterogeneity, whereas I^2^ values of 50% indicate a substantial level of heterogeneity. If possible, funnel plots will be used to assess the presence of potential reporting biases. A linear regression approach will be used to evaluate funnel plot asymmetry.

If the studies are too heterogeneous, then a narrative synthesis will be undertaken. For studies with qualitative evidence, a meta-synthesis approach will be used for the synthesis of the included studies.

#### 2.5.3 Missing data

In the case of missing data or unclear data (i.e., risk of bias unclear) deemed to possibly be important for this evaluation, we will attempt to contact the corresponding investigators of the article. If we cannot resolve the issues with the data after contacting the authors, we will do an analysis with the available data and discuss the possible impact of the missing data.

#### 2.5.4 Subgroup analyses

If sufficient data are available, we will perform the following subgroup analyses: specific details of the interventions (e.g., methodological strategy, components, and duration), research scenario (family participation, socioeconomic conditions), and risk of bias.

## 3 Discussion

The proposed systematic review and meta-analysis will present studies that evaluated the effects of school-based food and nutrition education interventions on adolescent health promotion.

Systematic reviews and meta-analyses show that interventions at school have positive effects or some potential for changes in school food consumption.^[6,8–10,25,26]^

However, it is important to emphasize that these studies were performed with child-adolescent binomial or only with the group of children. Recent review studies evaluating school-based interventions specifically for adolescents are scarce.

Two systematic reviews of food and nutritional education interventions for adolescents only evaluated the technology-based methodological strategy (the internet and social media platforms).^[12,13]^

School-based internet obesity prevention programs have apparently been successful in reaching high-risk students and changing behaviors in the short-term.^[12]^

Regarding the effectiveness of social media interventions in promoting positive changes in nutritional behaviors among adolescents, the current evidence shows that the increase in the intake of desirable food groups was more successful than decreasing unfavorable food habits.^[13]^

Another study with adolescents specifically investigated the potential role of fruit and vegetable consumption in cardiovascular health and concluded that the associations are inconsistent, probably due to heterogeneity in the methods used to assess and classify consumption and to define cardiovascular risk in adolescents.^[14]^

Finally, we found a review that points out that multi-strategy interventions can have significant impacts on the nutrition of adolescents when the nutrition education is theoretically based, facilitated by school staff in conjunction with parents and families and includes changes in the food environment from school. However, this review was restricted to studies conducted in developed countries, published from 2000 to 2014.^[15]^

In this context, we observed that school-based nutrition and nutritional education programs have significant results in children and when assessing the child-adolescent binomial. However, the results are scarce on the effect of different methodological strategies, specifically in the adolescence period. This protocol aims to overcome these limitations by quantitatively and qualitatively analyzing the effect of school-based nutrition education interventions on the promotion of adolescent health.

## Author contributions

**Conceptualization:** Gidyenne Christine Bandeira Silva de Medeiros, Grasiela Piuvezam.

**Data curation:** Gidyenne Christine Bandeira Silva de Medeiros, Kesley Pablo Morais de Azevedo, Victor Hugo de Oliveira Segundo, Ádala Nayana de Sousa Mata, Daniel Ángel Garcia, Clélia de Oliveira Lyra, Grasiela Piuvezam.

**Formal analysis:** Gidyenne Christine Bandeira Silva de Medeiros, Kesley Pablo Morais de Azevedo, Victor Hugo de Oliveira Segundo, Ádala Nayana de Sousa Mata, Grasiela Piuvezam.

**Investigation:** Gidyenne Christine Bandeira Silva de Medeiros, Karla Silveria Dias Pinheiro de Siqueira, Anny Karoliny Pinheiro Fernandes, Raquel Praxedes dos Santos, Débora Danielly Barros de Brito Trindade.

**Methodology:** Gidyenne Christine Bandeira Silva de Medeiros, Kesley Pablo Morais de Azevedo, Daniel Ángel Garcia, Victor Hugo de Oliveira Segundo, Ádala Nayana de Sousa Mata, Karla Silveria Dias Pinheiro de Siqueira, Grasiela Piuvezam.

**Project administration:** Gidyenne Christine Bandeira Silva de Medeiros, Clélia de Oliveira Lyra, Grasiela Piuvezam.

**Supervision:** Clélia de Oliveira Lyra, Grasiela Piuvezam.

**Writing – original draft:** Gidyenne Christine Bandeira Silva de Medeiros.

**Writing – review & editing:** Gidyenne Christine Bandeira Silva de Medeiros, Kesley Pablo Morais de Azevedo, Daniel Ángel Garcia, Victor Hugo de Oliveira Segundo, Ádala Nayana de Sousa Mata, Karla Silveria Dias Pinheiro de Siqueira, Anny Karoliny Pinheiro Fernandes, Raquel Praxedes dos Santos, Débora Danielly Barros de Brito Trindade, Clélia de Oliveira Lyra, Grasiela Piuvezam.

## Supplementary Material

Supplemental Digital Content
