# Supplementary material for: Protocol for systematic reviews of school-based food and nutrition education intervention for adolescent health promotion: Evidence mapping and syntheses
Source: Medicine (Baltimore). 2019 Aug 30;98(35):e16977. doi: 10.1097/MD.0000000000016977 (PMC6736443; doi:10.1097/MD.0000000000016977)
Supplement: Supplemental Digital Content [file medi-98-e16977-s001.docx]

**Appendix 1 -** Search strategies for each database.

| **Database** | **Search strategies** |
| --- | --- |
| MEDLINE/ PUBMED | (((((Adolescent) AND (Education or Teaching)) AND (Diet, Food, and Nutrition)) AND “School Health Services”) |
| EMBASE | (((adolescent OR teenager) AND education OR teaching) AND food OR nutrition) AND 'school health service' |
| SCOPUS | (Adolescent) AND (Education or Teaching) AND (Diet, Food, and Nutrition) AND (“School Health Services”) |
| ERIC | (Adolescent) AND (Education or teaching) AND (“School Health Services”) AND (Fruit or Vegetables or “Dairy products” or candy or “Fast Foods”) AND (“clinical trial")  Filter: Journal articles |
| SCIENCEDIRECT | (Adolescent) AND (Education) AND (Nutrition) AND (School Health Services) AND (“Clinical trial”)  Filter: Research articles. |
| WEB OF SCIENCE | (Adolescent) AND (Education) AND (Nutrition) AND (School) |
| COCHRANE | (“Adolescent”) AND (“Education”) AND (“Nutrition”) AND (“School”) |
| LILACS | (Adolescent) AND (Food AND Nutrition) AND (School) |
| ADOLEC | (Adolescent) AND (Food AND Nutrition) AND (School) |
